# Responsible Factors of Panic Buying: An Observation From Online Media Reports

**DOI:** 10.3389/fpubh.2020.603894

**Published:** 2020-11-05

**Authors:** S. M. Yasir Arafat, Sujita Kumar Kar, Vikas Menon, Angi Alradie-Mohamed, Srijeeta Mukherjee, Charanya Kaliamoorthy, Russell Kabir

**Affiliations:** ^1^Department of Psychiatry, Enam Medical College and Hospital, Dhaka, Bangladesh; ^2^Department of Psychiatry, King George's Medical University, Lucknow, India; ^3^Department of Psychiatry, Jawaharlal Institute of Postgraduate Medical Education and Research, Puducherry, India; ^4^School of Allied Health, Faculty of Health, Education, Medicine, and Social Care, Anglia Ruskin University, Chelmsford, United Kingdom; ^5^Department of Psychiatry, Maharaja Krushna Chandra Gajapati Medical College, Brahmapur, India

**Keywords:** panic buying, media report analysis, content analysis, COVID-19, pandemic

## Abstract

**Background:** Panic buying is an erratic human behavior that has been reported irregularly and episodically. There is a dearth of studies exploring the identifiable factors accounting for it. We aimed to identify the factors responsible for panic buying extracted from online media reports.

**Methods:** We scrutinized the media reports published in English discussing the different aspects of panic buying. We collected data until May 30, 2020, and searched the possible mentioned reasons responsible for panic buying.

**Results:** We analyzed a total of 784 media reports. The majority of the reports were found in Bing (18%), Ecosia (12.6%), Google (26.4%), and Yahoo (12.5%). Panic buying was reported in 93 countries. Among the 784 responses, a total of 171 reports did not explain the responsible factors of panic buying. Therefore, we analyzed the remaining 613 reports to identify the same. A sense of scarcity was reportedly found as the important factor in about 75% of the reports followed by increased demand (66.07%), the importance of the product (45.02%), anticipation of price hike (23.33%), and due to COVID-19 and its related factors (13.21%). Other reported factors were a rumor, psychological factors (safety-seeking behavior, uncertainty, anxiety reduction, and taking control), social learning, lack of trust, government action, and past experience.

**Conclusions:** The study revealed the responsible factors of panic buying extracted from media reports. Further, studies involving the individuals indulging in panic buying behavior are warranted to replicate the findings.

## Introduction

Panic buying (PB) is an erratic human behavior that has been reported irregularly and episodically; however, PB has been reported since long before and appeared especially during a major emergency event (1–3). It has been noticed and reported in online media during this COVID-19 pandemic in several countries in the world (1, 4, 5). It has been explained as “the phenomenon of a sudden increase in buying of one or more essential goods in excess of regular need provoked by adversity, usually a disaster or an outbreak resulting in an imbalance between supply and demand” (4). A group of authors speculatively described it as a manifestation of underlying conflict and uncertainty during the pandemic, a way of coping with a stressful condition, gaining control, and social pressure to conform to alike behaviors (5). Theoretically, few mental processes have been discussed such as the perception of scarcity of necessary goods, way to gaining control, uncertainty, insecurity, herd behavior, primitive behavior, media influence, and lack of confidence in authorities (6). A recent systematic review also revealed some psychological factors responsible for PB mentioned such as (a) perceived threat and scarcity of the goods, (b) fear of the unknown resulting from negative emotions and uncertainty, (c) coping behavior such as anxiety reduction and gaining control, and (d) social–psychological issues (7). Singh and Rakshit mentioned PB as herd behavior (8) and Tsao et al. mentioned supply chain disruption (9). Again, Chen et al. (2) described that disturbed judgments resulting from improper information during a crisis are responsible for PB where authors tried to explain it with economics and psychology theories. The endowment effect and commodity and prospect theories have been proposed to explain PB based on economics (2). Additionally, the authors hypothesized three mental processes mentioning autonomy, relatedness, and competence as attributing factors for PB (2).

Although newer studies are coming out, there is a dearth of empirical studies exploring the identifiable factors responsible for it. However, there are also challenges to studying the phenomenon systematically as it is erratic, irregular, episodic, sudden, unpredictable, and mostly happens during emergency situations (10). Interestingly, PB is a newsworthy issue and has been frequently reported (1, 4). Therefore, we aimed to evaluate the responsible factors of PB extracted from online media reports.

## Materials and Methods

### Study Setting and Data Collection

This was a prospective analysis of 18 internet search engines (Table 1), all of which were identified a priori by the team of investigators. Three different investigators equally divided the search engines and simultaneously carried out the search using only the term “panic buying”; no combination was used to minimize missing media report that discussed PB. We scrutinized the media reports published in English discussing the different aspects of PB with special attention to attributable factors of it. We searched and collected data from 1 to 31 May 2020 in *Google Form*. Data were collected from any report from the media covering the blogs, personal views, opinions, and news. We excluded social media posts as social media posts are more of personal views and very often emotionally biased. People may post/repost things that are trending just to appear active online without really understanding its impact. Conformity bias and groupthink could act as other sources of potential biases. Subsequently, two other investigators did cross-checks of the data and data cleaning. Duplications were checked and removed by tracing the date of publication and title of the reports. Data collectors were well trained through frequent Zoom meetings by the team of investigators before starting the study. Doubts on whether to include a report were sorted out by mutual discussion with a senior author (SMYA, SKK). In case they were unable to reach an agreement, the issue was resolved by involving all authors in a group discussion. The above methodology was adapted from previous media-based studies on PB that used a single keyword search strategy (1, 4).

**Table 1:** Search details.

**Search engine**	***n***	**%**
Aol.com	28	3.6
Baidu	6	0.8
Bing	141	18.0
Duckduckgo	36	4.6
Ecosia	99	12.6
Exalead	11	1.4
Excite	13	1.7
Gigablast	4	0.5
Google	207	26.4
Lycos	3	0.4
Mojeek	8	1.0
Qwant	9	1.1
Startpage.com	52	6.6
Swisscows	28	3.6
Webcrawler	13	1.7
Yahoo	98	12.5
Yandex	14	1.8
Yippy	14	1.8
Total	784	100

#### The Instrument

Based on the existing literature (3, 5, 6) and our previous works (1, 4), the team formulated the questionnaire through the Zoom meeting. The instrument had two sections consisting of (a) the identification section and (b) the attributed factors section. The identification section comprised the name of the country from where the report was published, the name of the country to which the report referred, type of newspaper, name of the newspaper, dates of publication, and the primary scarce object for PB. All the reports were scrutinized by careful reading, and all the mentioned attributable factors were identified and documented in the second section. We aimed to identify all the possible factors and the majority of the reports discussed several factors.

#### Statistical Analysis

We used simple descriptive statistics (frequency and percentages) to depict extraction such as the number of relevant reports identified from different search engines as well as various reasons for PB. A word cloud analysis was used to summarize search results in terms of frequency of PB reports from different countries.

#### Ethics Statement

The study was conducted complying with the declaration of Helsinki (1964). As we analyzed the publicly available media reports, no formal ethical approval was obtained.

## Results

**Figure 1:**
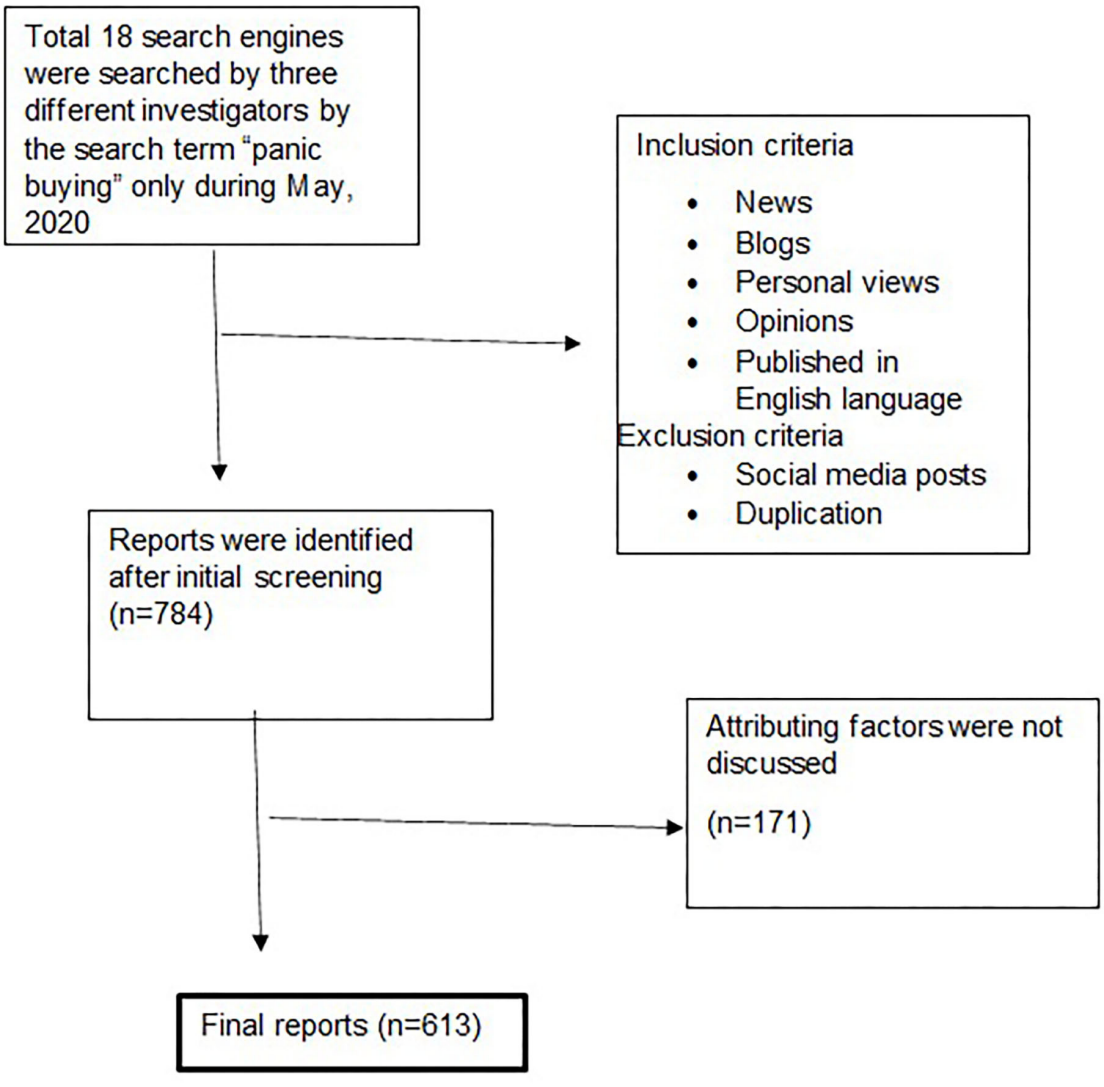
Flow chart showing study sample selection.

**Figure 2:**
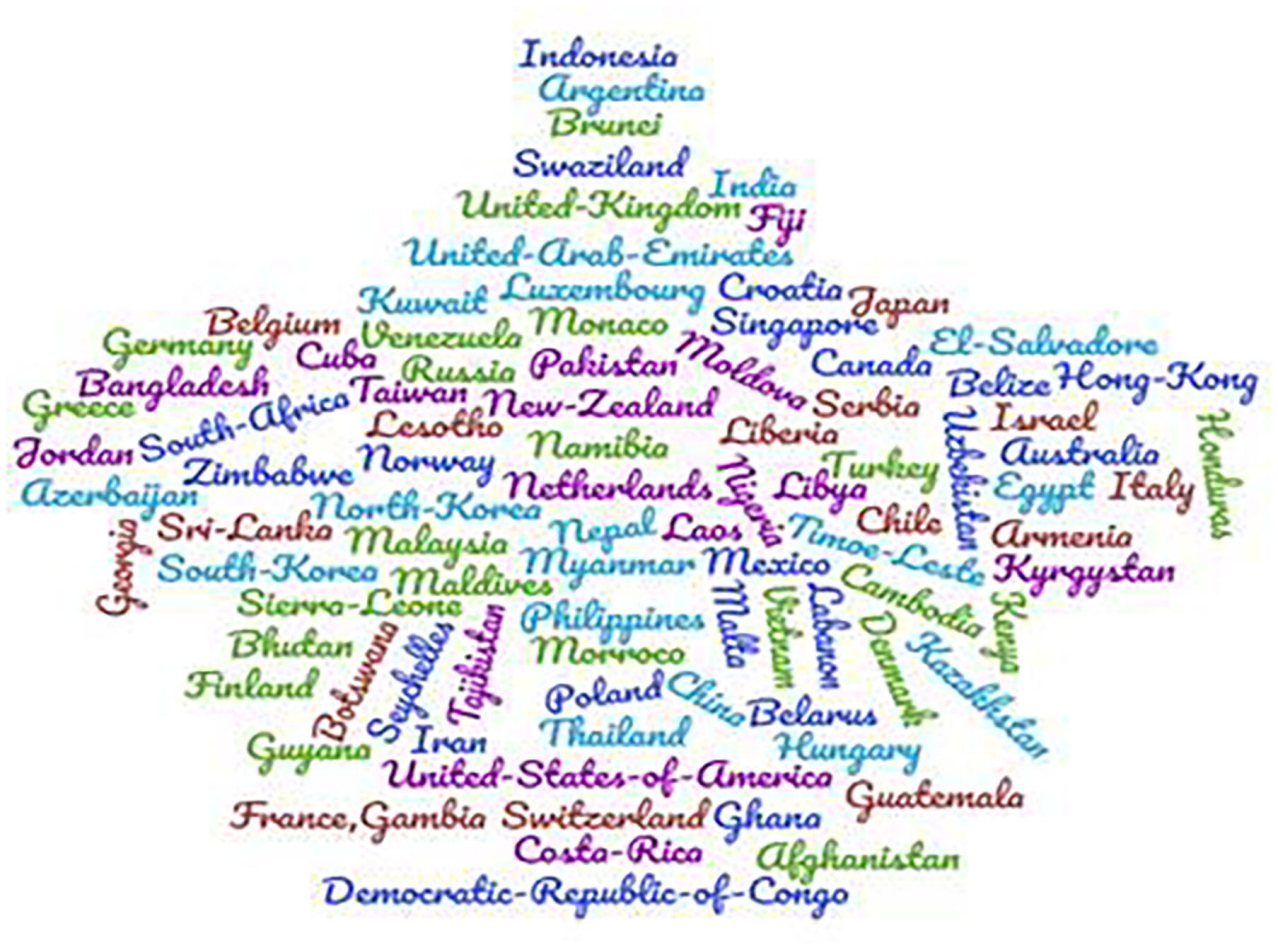
Word cloud showing countries reporting panic buying behavior.

We analyzed a total of 784 media reports (Figure 1). The majority of the reports were found in Bing (18%), Ecosia (12.6%), Google (26.4%), and Yahoo (12.5%) (Table 1). PB was reported in 93 countries (Figure 2). Among the 784 responses, a total of 171 reports did not explain the responsible factors of PB. Therefore, we analyzed the rest 613 reports to identify the responsible factors of PB. We considered extracting as many as possible attributing factors from the contents. Therefore, a single report had multiple responses when the factors were segregated. A sense of scarcity was reportedly found as the important responsible factor of PB that was found in about 75% of the reports (*n* = 456) followed by increased demand (66.07%), the importance of the product (45.02%), anticipation of price hike (23.33%), and due to COVID-19 and its related factors (13.21%). The rumor was mentioned as responsible factors in 53 reports (8.65%). We considered safety-seeking behavior (*n* = 5), uncertainty (*n* = 6), anxiety reduction (*n* = 24), and taking control (*n* = 15) as psychological factors, which constituted 8.16% (*n* = 50) of reports. Other reported factors were social learning, lack of trust, government action, and past experience (Table 2).

**Table 2:** Reasons of panic buying (*n* = 613).

**Reasons of PB**	***n***	**%**
Scarcity	456	74.39
Increased demand	405	66.07
Necessary goods	276	45.02
Anticipated price hike	143	23.33
COVID-19, lockdown, planned,	81	13.21
Rumor	53	8.65
Psychological	50	8.16
Social learning	15	2.45
Lack of trust	6	0.98
Government's action	6	0.98
Past experience	3	0.49
Total	613	100

## Discussion

PB is a contemporary issue with a dearth of empirical studies regarding the responsible factors behind the behavior. We aimed to evaluate the responsible factors of PB extracted from online media reports. The study revealed that PB has been reported in 93 countries. Previous studies reported the distribution of the countries; however, none of the studies mentioned such wide distributions (1, 2, 4, 5).

The study revealed several responsible factors, i.e., a sense of scarcity, increased demand, the importance of the goods, the anticipation of price hike, COVID-19 pandemic, rumor, safety-seeking behavior, uncertainty, anxiety reduction, taking control, social learning, lack of trust, government action, and past experience as the responsible factors for PB (Table 2). A high rate attribution was mentioned in reports among few factors such as perceived scarcity (75%), increased demand (66%), the importance of the product (45%), and anticipation of price hike (23%). The COVID-19 pandemic and related issues were attributed to about 13% of the reports. The rumor was mentioned as responsible factors in 53 reports (8.65%). A recent systematic review identified the factors grossly in four domains, namely, perception, fear of the unknown, coping strategy, and psychosocial factor (7). The authors also subdivided the gross areas into different parts. Others mentioned the responsible factors as a mismatch between routine work and uncertainty, coping strategy, gaining control, social learning, and supply chain disruption (5, 8, 9). It can also be due to any perceived or real external threat and/or own nervousness (11).

Perceived scarcity has been mentioned repeatedly by different groups of authors as an important responsible factor for PB (2, 6, 7). However, perception of threat and/or perception of risk have also been mentioned as a factor that has not been found in the current study (3, 7). We speculate that there might have been some overlaps between the perception of risk and our included psychological factor, which covers safety-seeking behavior, uncertainty, anxiety reduction, and taking control.

The second most important attributing factor has been identified as increased demand that was mentioned in about 66% of the responses. It is quite interesting, and it could be a result rather a causative factor because in a normal equilibrium, the demand should not be increased until an adverse and/or precipitating event occurs. It can be explained by taking consideration of the proposed definition by Arafat et al. (4). The phenomenon starts with a sudden increase in buying precipitated by adversity, usually a disaster or an outbreak resulting in a shortage of supply (4). The supply shortage can precipitate the rise of demand (9). Furthermore, the shortage of necessary goods is widely circulated by the media, which in turn creates insecurity, uncertainty, and more PB. Therefore, increasing demand can be explained by the precipitating events and results of the dissemination of shortage news.

The third important identified factor is the importance of the product that has been attributed to about 45% of the reports. It is quite plausible because PB mostly happens in cases of essential goods that are supposed to be used in the immediate future. Previous studies also mentioned similar factors (4, 5).

The fourth important identified factor is the anticipation of the price hike that was attributed to about 23% of the reports. A complex interaction should be warranted in case of anticipation of price hike, because the precipitating event such as the COVID-19 pandemic, perception regarding the supply chain, media propagation, rumors, and personal experience may have interaction to bear the perception of price hike (2).

Other responsible factors are the COVID-19 pandemic, rumor, safety-seeking behavior, uncertainty, anxiety reduction, taking control, social learning, lack of trust, government action, and past experience as the responsible factors for PB (Table 2). All the factors could explain the previous explanations (2–7, 12–14).

**Figure 3:**
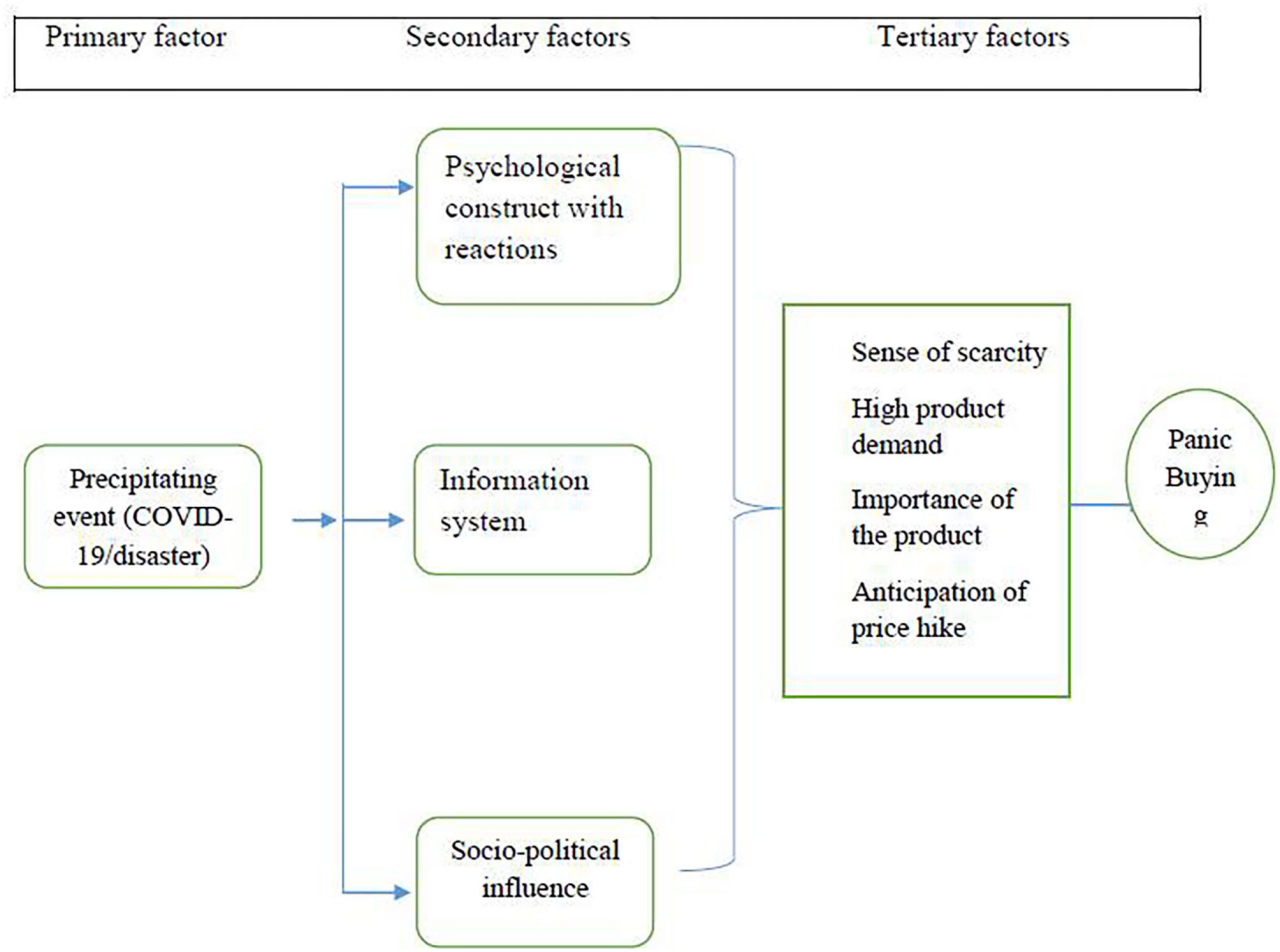
Causal model of panic buying.

Here, we theorized the adverse or precipitating event as the primary causative factor; psychological construct with reactions, social structure, and information system are the secondary factors; and subsequently, other factors such as sense of scarcity, increased demand, the importance of the product, and anticipation of the price hike are the tertiary factors (Figure 3). There is a complex constant interaction between the primary, secondary, and tertiary factors.

### Strengths of the Study

There is a dearth of empirical studies exploring different aspects of PB. To the authors' best knowledge, this is the first empirical study exploring the responsible factors of the behavior.

### Limitations

The current study has several limitations. Everyone should be aware of the scientific quality of data as we analyzed any sort of media reports that may not be considered as scientific data. We also included only media reports published in English and excluded reports published in other languages. We only searched with a single keyword, “panic buying,” without considering the synonyms, which may reduce the number of reports.

## Conclusion

The study revealed the factors responsible for PB extracted from media reports, which include a sense of scarcity, increased demand, the importance of the product, the anticipation of price hike, COVID-19, rumor, psychological factors (safety-seeking behavior, uncertainty, anxiety reduction, taking control), social learning, lack of trust, government action, and past experience. The extracted factors can be theoretically explained based on the previous reports revealing a complex interaction among a precipitating stimulus, personality construct, and environmental influences. Practically, adequate actions targeting the reasons could be beneficial for the prevention of PB. Preparedness for future episodes should have a special focus on the identified factors to reduce the panic among the general population. Further, empirical studies involving the individuals indulging the PB behavior are warranted to replicate the findings and/or nullify it. Qualitative studies could be a potential option to explore the psychological aspects responsible for the behavior.

## Data Availability Statement

The raw data supporting the conclusions of this article will be made available by the authors, without undue reservation.

## Ethics Statement

The study was conducted complying with the declaration of Helsinki (1964). As we analyzed the publicly available media reports, no formal ethical approval was obtained.

## Author Contributions

SA contributed to the idea, concept, design, data analysis, and writing. SK and RK contributed to the concept. VM contributed to concept, design, and writing. AA-M, SM, and CK contributed to the data enumeration. All authors contributed to the article and approved the submitted version.

## Conflict of Interest

The authors declare that the research was conducted in the absence of any commercial or financial relationships that could be construed as a potential conflict of interest.
